# The effect of sorghum resistance resistant starch‐mediated equol on the histological morphology of the uterus and ovaries of postmenopausal rats

**DOI:** 10.1002/fsn3.1670

**Published:** 2020-06-17

**Authors:** Yun‐Fei Ge, Chun‐Hong Wei, Wei‐Hao Wang, Long‐Kui Cao

**Affiliations:** ^1^ College of Food Science Heilongjiang Bayi Agricultural University Daqing China; ^2^ National Coarse Cereals Engineering Research Center Heilongjiang Bayi Agricultural University Daqing China

**Keywords:** intestinal flora, menopause, ovary, resistant starch, sorghum, uterus

## Abstract

Equol is a metabolite of daidzein and has a higher biological activity than daidzein. Equol, combined with estrogen receptors, can reduce the incidence of diseases such as cardiovascular disease, osteoporosis, and breast cancer; more effectively alleviate the symptoms of perimenopausal syndrome; and improve age‐related decline of the uterus and ovaries. Research has shown that food composition can greatly affect the formation of equol in the intestinal tract. In the intestines, the content of nonstarch polysaccharides that can stimulate fermentation is high, thereby allowing intestinal bacteria to quickly and completely transform the daidzein into equol. This study used Sprague Dawley (*SD*) rats as a model, where menopause was established through direct intragastric administration of formistan. In the 6‐week‐long experiment, intragastric administration of RS while feeding bean pulp reduced the body weight of postmenopausal rats, reduced the efficiency of feed utilization of rats, and increased the weight of organs such as the uterus and ovaries. Routine blood indexes showed that no adverse reactions were produced by intragastric administration of RS. 16s rDNA sequencing further verified *Lactobacillus* and *Clostridium* XIVa, as the bacteria that converted daidzein into equol.

## Graphical abstract

In this study, the evaluation of equol content is important for the changes of uterine and ovarian morphological tissues in menopausal rats under the condition of adding resistant starch. The information obtained has a certain repairing effect on uterine ovarian atrophy in postmenopausal women. Resistant starch can regulate the structure of the intestinal flora and metabolize daidzein in soy products to produce equol to exert higher biological activity and to act as a substitute for estrogen in postmenopausal women. Therefore, in the development of functional foods, the combination of resistant starch and soybean products can be selected.

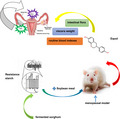

## 1 INTRODUCTION

Advances in science and technology have resulted in a rapidly growing aging population. With the development of human society and the progress of science and technology, the aging of the population has become a prominent problem at present. According to WHO, the world's elderly population is predicted to exceed 578 million in 2000, of which 70% will be elderly women (Ding, 2006). Women spend one third of their lifetime in perimenopausal and postmenopausal stages, during which they gradually show symptoms due to the lack of estrogen, thereby negatively affecting their health and quality of life. Uterine and ovarian tissue atrophy are the major causes of perimenopausal syndrome. Data show that low levels of estrogen postmenopause cause hectic fever, insomnia, night sweats, mood swings, and other symptoms, and that long‐term estrogen deficiency may cause cardiovascular diseases, osteoporosis, and urogenital diseases (Luo, 2003). Therefore, delaying the natural aging of the genital rachis is of great value for improving the quality of life for perimenopausal women.

Equol, as a phytoestrogen, has attracted extensive attention because of its strengths compared with its precursor daidzein: It has greater beneficial biological activities, increased appetency for estrogen receptors, higher antioxidant activity, and stronger anti‐cancer effects. (Kostelac, Rechkemmer, & Briviba, 2003; Muthyala, Ju, & Sheng, 2004). Research suggests that food composition greatly affects the formation of equol in the intestinal tract. In the presence of a high content of nonstarch polysaccharides that can stimulate fermentation, intestinal bacteria can quickly and completely convert daidzein into equol, while in cases of low‐carbohydrate content, it is almost impossible to produce equol (Rowland, Wiseman, Sanders, Adlercreutz, & Bowey, 2000). That is, dietary fibers or nonstarch polysaccharides can effectively promote the growth or activity of the colonic bacteria that produce equol (Frankenfeld, 2011). Sorghum, as the fifth largest grain crop, is widely cultivated because of its high yield and stress resistance (Wang & Li, 2006). As a type of grass, sorghum has a high‐carbohydrate content of up to 80%, making it a rich source of resistant starch (RS), a novel type of dietary fiber. RS has good enzymolysis resistance and cannot be digested and absorbed in the small intestine. It is fermented in the large intestine and produces short‐chain fatty acids, which improves the intestinal environment and adjusts the composition of the intestinal flora. At present, it is widely believed that intestinal physiology, host genotype, and dietary composition are the main factors affecting an individual's ability to convert daidzein into equol (Huang, 2010). Therefore, the novelty of this study is in the improvement of the intestinal flora through RS to convert daidzein from bean pulp to equol to ultimately reduce the severity of perimenopausal symptoms in rats.

These experiments showed that when sorghum was naturally fermented for eight days, the content of amylose and the retrogradation value peaked, the RS was easier to prepare, and the fermentation characteristics of the obtained RS were higher. Therefore, natural fermentation of sorghum was carried out in this experiment, and RS was prepared using the pressure‐heat compound enzyme method. In vivo experiments were conducted to explore the effect of RS‐mediated intestinal flora on the morphology of ovarian and uterine tissues of menopausal rats.

## 2 MATERIALS AND METHODS

### 2.1 Experimental material and equipment

#### 2.1.1 Experimental material

Sorghum: Sorghum R from Linyi, Shandong. Hydrochloric acid (analytically pure), sodium oxide (analytically pure), potassium bromide (spectrographic grade), high‐temperature‐resistant α‐amylase (1,400 u/g), pullulanase (1,000 ASPU/g), and high‐amylose corn starch were made by Damao Chemical Reagent Factory, Tianjin, China. Glucan standards and estradiol standards were purchased from Sigma. Vancomycin, neomycin, metronidazole, and penicillin were made by Qingdao High‐Tech Park Haibo Biotechnology Co., Ltd. Sprague Dawley rats were from Changchun Yisi Experimental Animal Technology Co., Ltd. Feed including bean pulp and feed excluding bean pulp was from Biotech HD. Distilled water was produced by the laboratory.

### 2.2 Instruments and equipment

Dgg‐9053A electrothermal blowing dry box by Senxin Laboratory Instrument Co., Ltd., Shanghai; MJ‐10A flour mill by Puheng Information Technology Co., Ltd.; LS‐3781L‐PC autoclave by Panasonic; BA210T microscope by Jiwei Medical Device Co., Ltd.

### 2.3 Preparation of samples

Sorghum was naturally fermented for 8 d. Sorghum was mashed and screened with an 80 sieve and soaked in 0.3 g/100 ml NaOH at 1:3 g/ml for 3 hr followed by centrifuging for 10 min at 3256 *g*. The supernatant and upper tawny substances in the sediment were removed, washed with clean water four times, and centrifuged until the starch slurry became white. The pH of the starch slurry was adjusted with 1 mol/L HCl to 7.0, centrifuged, dried at 30°C, and screened with a size 80 sieve to obtain fermented sorghum starch (Xu, Cheng, & Zhao, 2007.).

Fermented sorghum starch was used to prepare 10% starch milk. Starch milk was autoclaved, pressure‐heated for 15 min at 115°C, cooled down to 40°C, and pH adjusted to 4.5. Then, 3 U/g pullulanase was added and treated for 8 hr at 40°C, followed by enzyme deactivation for 15 min at 95°C. Samples were cooled to room temperature and kept at 4°C for 12 hr (Bao, 2017). Retrograded starch was removed, dried, and prepared into 20% RS starch milk. The pH was adjusted to 5.4 and 2 ml of 1% high‐temperature‐resistant α‐amylase was added and vibrated in 90°C water for 1.5 hr. The starch paste decomposed into monosaccharides and oligosaccharides through enzymolysis and was then heated in boiling water for 10 min followed by the addition of 4 times the volume of 95% ethyl alcohol to dissolve the monosaccharides and oligosaccharides. The alcohol precipitated for 4 hr before being centrifuged for 20 min at 2442 *g*. The supernatant was removed and 10 ml of 95% ethyl alcohol was added to wash the sediment 2–3 times followed by drying at 50°C to a constant weight, mashed, and screened with a size 100 sieve to get fermented sorghum RS (Wang, 2013).

### 2.4 Diet and animals

Seventy Sprague Dawley rats, three months of age, were purchased from Changchun Yisi Laboratory Animal Technology Co., Ltd. Each of them weighed 190 ± 10 g and were maintained in the facility at room temperature (24 ± 2°C) with 45%–65% humidity. Animals were divided into eight groups, blank group (NC), model group (MC), RS group (RS), low‐dose RS group (RSL), medium‐dose RS group (RSM), high‐dose RS group (RSH), positive control group (PC), and fecal microbiota transplantation group (FMT), and each rat was fed in single cages. Menopause was established by gavage of Formestane every day at 9 a.m., at a dosage of 50 mg/kg per animal, except for the blank group (NC). During the modeling, the animals had ad‐libitum access to water and were fed a bean‐free diet. An ELISA kit was used to determine the concentrations of follicle‐stimulating hormone and estradiol in the urine of the rats. The aim of this test was to determine the stage of menopause. After the rats were determined to be in the menopausal state, drugs such as resistant starch were administered. The determination of the concentration of resistant starch was based on the recommended daily intake of healthy humans; it was processed and dissolved with an ultrasonic processor. The design of test drug delivery is shown in Table 1. The experimental diet composition was designed with reference to AOAC synthetic feed formula (AIN‐93) for experimental rats. Soybean‐free diets were prepared by replacing bean pulp with casein (Reeves, Nielsen, & Fahey, 1993). Animal feeding and treatment were in accordance with the provisions of the Chinese Association for Laboratory Animal Sciences (CALAS), and all animals were cared for in strict accordance with animal use guidelines.

**TABLE 1:** Grouping and feeding of animals

Group	Feed	Repeat (number)	Treatment (day)	Time (week)
NC	Ordinary feed	9	Saline 2 ml	6
MC	Ordinary feed	9	Saline 2 ml	6
RS	No soy feed	9	RS 1.8 g/kg	6
RSL	Ordinary feed	9	RS 0.9 g/kg	6
RSM	Ordinary feed	9	RS 1.8 g/kg	6
RSH	Ordinary feed	9	RS 2.7 g/kg	6
PC	Ordinary feed	9	high‐amylose maize starch 1.8 g/kg	6
FMT	Ordinary feed	7	Feces of rats with the highest level of equol production 0.1g	6

Fecal microbiota transplantation (FMT) (Gong et al., 2018): Before the experiment, vancomycin (1 g/L), neomycin (1 g/L), metronidazole (1 g/L), and penicillin (500 g/L) were dissolved in the drinking water of rats. After one week of interference, the intestinal flora of rats was cleared to balance and unify the flora environment in vivo. Fresh feces (100 mg) from the donor rats with the highest content of equol were selected and mixed with 1 ml sterilized normal saline in a centrifuge tube and left standing for 10 min. An aseptic syringe was used to transfer the supernatant to feed the rats through intragastric administration.

During the 6‐week‐long experiment, urine was collected every week, and the weight and food intake of the rats were recorded. At the end of the experiment, feces samples were collected for immediate freezing and storage in −80°C. After fasting overnight, the rats were euthanized, and the eyes were removed for blood samples. The uterus, ovaries, thymus, and spleen were harvested and weighed. After weighing, the uterus and ovaries were stored in a formaldehyde solution until histomorphological analysis.

### 2.5 Body weight of rats: determination of weight gain and efficiency of feed utilization

During the experiment, body weight and food intake of each rat were recorded weekly, and the feed efficiency of the rat was recorded according to the following formula (1):

$$\text{utilization}=\frac{M_{1}{\;-\;M}_{0}}{M}\times 100\%$$

Where: M is the total food intake of the rat during the experiment;M_0_ is the initial weight of the rat;M1 is the weight of the rat at the end of the experiment.

### 2.6 Determination of routine blood indexes

After the rat was weighed and blood was taken from the eye post‐removal, the blood was collected into a blood vessel with an EDTA‐K2 anticoagulant tube. Blood indexes from freshly collected blood were determined in an immobilized blood cell analyzer.

### 2.7 Diversity of the intestinal flora of postmenopausal rats

#### 2.7.1 Sample pretreatment

A total of 200 mg of feces were added into a centrifuge tube with 1 ml of 70% ethyl alcohol, vibrated to mix, and centrifuged for 3 min at 8000 *g* at room temperature. The supernatant was removed and 1 ml of phosphate‐buffered saline was added, vibrated to mix, and centrifuged for 3 min at 8000 *g* at room temperature. The supernatant was removed, and the tubes were inverted for 1 min or until all remaining liquid was removed. Sample tubes were dried at 55°C for 10 min to remove residual ethyl alcohol.

#### 2.7.2 DNA extraction

Bacterial DNA from the feces of postmenopausal rats was extracted with a DNA kit (Nanjing Jiancheng Bioengineering Research Institute), and extraction was performed according to manufacturer's protocol.

#### 2.7.3 PCR amplification and annealing temperature control of the DNA fragment of bacterial 16S rDNA

A kit was adopted for PCR amplification of the DNA fragment of bacterial 16S rDNA. Group operation followed the instructions of the kit. The PCR primer integrated the V3‐V4 universal primer of the sequencing platform MiSeq. 341F primer: CCCTACACGACGCTCTTCCGATCTG(barcode) CCTACGGGNGGCWGCAG. 805R primer: GACTGGAGTTCCTTGGCACCCGAGAATTCCA GACTACHVGGTACT. GACTACHVGGGTACTAATCC.

#### 2.7.4 Quantified mixture

The DNA reclaimed by detection kit pairs was accurately quantified to facilitate sequencing after mixing at a 1:1 ratio. From the balanced mix, 10 ng of DNA was taken for each sample and 2 pmoL of DNA was used for sequencing. Sequencing was completed by Sangon Biotech.

### 2.8 Determination of equol and daidzein contents

Fresh urine was collected, and the equol and daidzein content were determined by ELISA (Shanghai Fanke Industrial Co., Ltd.) The experiment was performed according to manufacturer's protocol.

The log Koc of the equol content:daidzein content, that is log10 (equol/daidzein), was used to represent the equol production capacity of postmenopausal rats (Setchell & Cole, 2006).

### 2.9 Histological analysis

Uterus and ovary tissues of postmenopausal rats were soaked in formaldehyde solution, fixed with paraffin, and stained with hematoxylin and eosin to make routine sections. The morphology of the liver was observed using a BA210T microscope equipped with a camera.

### 2.10 statistical analysis

Excel 2016 and SPSS 19.0 software were used for statistical analysis of the data; Origin 8.0 software was used for drawing processing; the data were measured in triplicate to obtain the average value. The results are presented as the mean ± *SD*. Group differences were analyzed using Tukey's post hoc test, and the data were measured by two‐way repeated‐measures analysis of variance (ANOVA).

## 3 RESULTS AND DISCUSSION

### 3.1 Effect of RS‐mediated intestinal flora on the body weight, feed consumption, and efficiency of feed utilization of postmenopausal rats

The effect of RS‐mediated intestinal flora on the weight and efficiency of feed utilization of postmenopausal rats is shown in (Table 2). Following menopause modeling, the weight of rats in the experimental groups, including the model group, was significantly higher than that of the untreated control group, which suggests that estrogen deficiency was an important factor in causing obesity phenotypes among postmenopausal rats. Intragastric administration of RS and supply of feed not containing bean pulp resulted in rats with body weight lower than that of the model group. This suggests that RS has an effect on the increase in rat body weight, because of which the feed consumption and efficiency of feed utilization were decreased. This finding can be explained by the fact that RS was not digested in the small intestine, thus not producing any energy, resulting in a long gastrointestinal emptying time which thereby intensified the satiety of the animal (Chen, Gao, & Wang, 2005). In the case of administration of RS of equal amounts in conjunction with feed containing bean pulp, the rats had significantly different body weight from the model group, which suggests a stronger effect on weight loss in postmenopausal rats. Feed efficiency reflects the digestion, absorption, and utilization of food in experimental animals. Comparing the feed efficiency of Group RSL and Group RS, Group RSL had a significantly lower feed efficiency than Group RS (*p* < .05). Group RSL showed a greater effect on weight loss by reducing the efficiency of feed utilization. This indicates that in conjunction with bean pulp, RS improved the structure of the intestinal flora as a fermentation substrate, converting the daidzein in the bean pulp into equol. The production of equol would have resulted in improved biological functions compared with just RS and could more effectively control the weight gain caused by long‐term massive food intake. The fecal microbiota transplantation reduced the weight of postmenopausal rats further, suggesting that under the effect of intestinal flora, the equol converted from daidzein participated in fat metabolism and enhanced the activities of related enzymes (Zhou & Hu, 2005), thus causing the decrease in rat body weight, as well as weight gain and feed consumption rate.

**TABLE 2:** Effect of RS‐mediated intestinal flora on body weight, feed consumption, and efficiency of feed utilization of postmenopausal rats

Group	The body weight of rat/g							Weight gain (g)	Feed consumption (g)	Feed efficiency (%)
	0 week	1 week	2 weeks	3 weeks	4 weeks	5 weeks	6 weeks			
NC	250 ± 14b	266 ± 17b	287 ± 21c	304 ± 16c	323 ± 22c	351 ± 24d	369 ± 13d	119 ± 1c	2,364 ± 123e	5.03 ± 0.008d
MC	312 ± 17a	352 ± 14a	389 ± 21a	427 ± 23a	463 ± 18a	502 ± 11a	554 ± 16a	242 ± 1a	4,257 ± 89a	5.68 ± 0.011a
RS	315 ± 21a	354 ± 16a	387 ± 12a	422 ± 13a	447 ± 22a	474 ± 24ab	519 ± 15b	204 ± 6b	4,158 ± 104a	4.91 ± 0.057e
RSL	318 ± 15a	358 ± 21a	391 ± 22a	407 ± 14a	439 ± 13a	481 ± 25ab	521 ± 19b	203 ± 4b	3,557 ± 114b	5.71 ± 0.035a
RSM	317 ± 14a	342 ± 23a	377 ± 17ab	403 ± 22a	438 ± 16a	463 ± 24c	498 ± 19b	181 ± 5b	3,385 ± 95b	5.34 ± 0.053c
RSH	314 ± 18a	339 ± 14a	351 ± 09b	371 ± 11b	396 ± 14b	421 ± 15c	446 ± 16c	132 ± 2c	3,121 ± 106c	4.23 ± 0.019f
PC	311 ± 16a	329 ± 13a	347 ± 15b	354 ± 11b	368 ± 18b	395 ± 14c	415 ± 14c	124 ± 2c	2,924 ± 91d	3.56 ± 0.022g
FMT	321 ± 13a	347 ± 18a	378 ± 14ab	421 ± 11a	459 ± 17c	485 ± 14a	524 ± 16a	138 ± 4c	2,462 ± 87e	5.61 ± 0.046b

Note

Data are shown as mean ± *SD* (*n* = 7/9). Values with different letters in columns are significantly different (*p* < .05) from each other.

### 3.2 Effect of RS‐mediated intestinal flora on the viscera weight of postmenopausal rats

**FIGURE 1:**
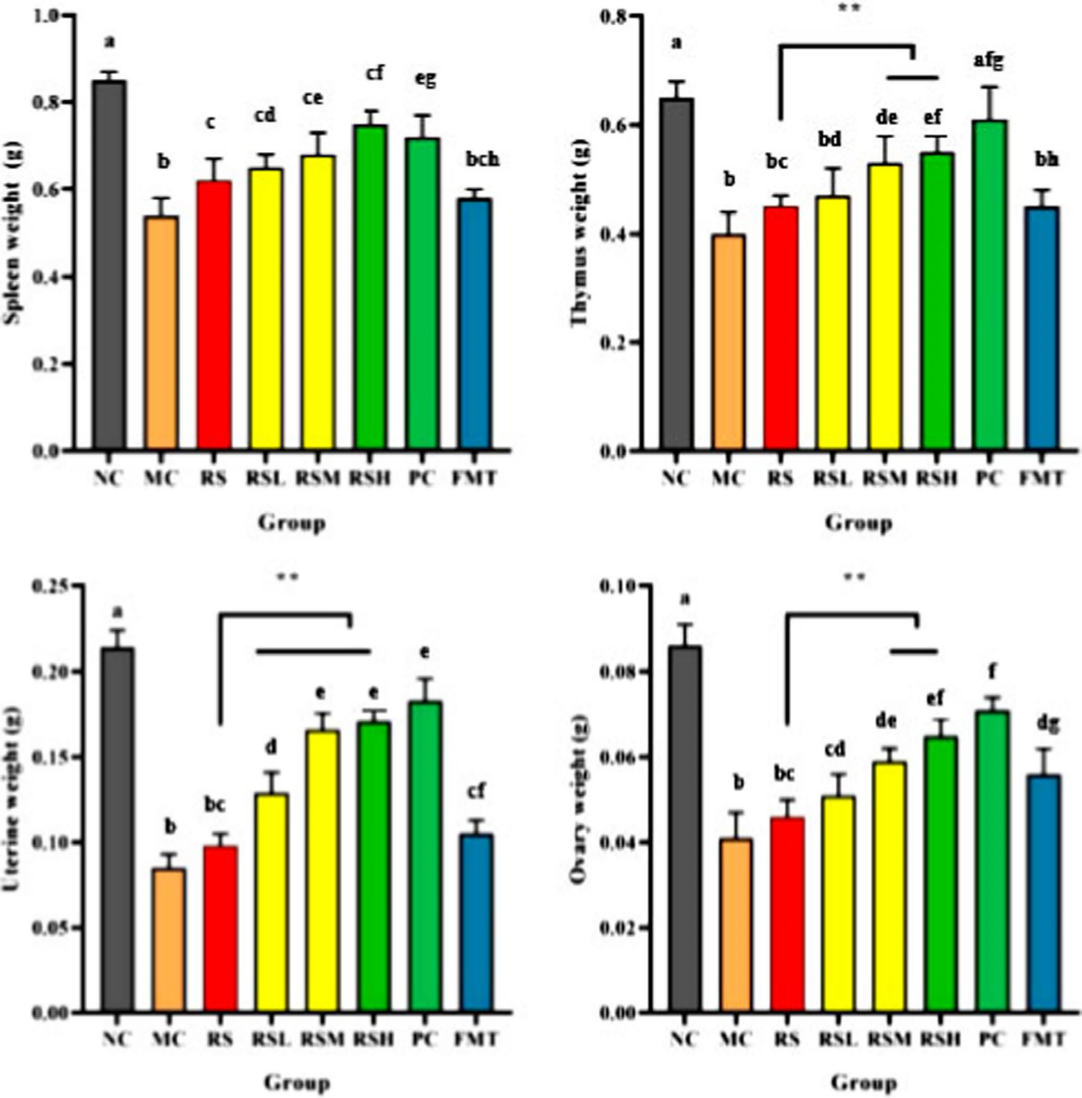
Effect of RS‐mediated intestinal flora on the viscera weight of postmenopausal rats. Values with different letters in columns are significantly different (*p* < .05) from each other; significance was determined by ANOVA test. *p < .05, **p < .01, ***p < .001

Compared with the untreated control group, the weight of the spleen and thymus decreased significantly in the model group (Figure 1), indicating that immune organs such as thymus and spleen shrank significantly after menopause. Menopause led to a gradual decrease in growth‐promoting hormones secreted by the hypothalamus, which led to a decrease in the secretion of endocrine hormones from the pituitary gland and target gland, thus further leading to insufficient immune functions and promoting the aging process of the body (Hirokawa, Utsuyama, Kasai, & Kurashima, 1992). Compared with the model group, each drug group could increase the weight of spleen, uterus, and other organs to a certain extent, and alleviate the failure of immune organs caused by estrogen deficiency. Compared with the control group, the weight of the uterus and ovaries in the rats after modeling decreased, reflecting the decreased reserve function of the uterus and ovary, and atrophy. These data confirm that menopause caused the change in weight and morphology of these organs in the rats. After drug treatment, the weight of organs in each group was significantly higher than that in the model group and close to that of the control group. Among them, Group RS had little influence on the weight of the uterus and ovaries, which indicates that RS had a weak effect on the improvement of uterus and ovary However, in the case of adding bean pulp to the feed, through intragastric administration of RS at different proportions, the weight of viscera was significantly increased. Group RSH and Group PC had no significant difference in the improvement of viscera. This indicates that RS had a remarkable effect in alleviating symptoms in the uterus and ovaries when bean pulp was added. This is due to the presence of high‐concentration polysaccharide fermentation substrates, which the intestinal flora would use to convert daidzein bean pulp into its metabolite equol with higher phytoestrogen properties (Frankenfeld, 2011) so as to improve the weight of the uterus and other organs. FMT further showed that the key to regulating the production of equol by RS was intestinal flora.

### 3.3 Effect of RS‐mediated intestinal flora on the routine blood indexes of postmenopausal rats

Various diseases and changes in physical conditions can lead to changes in routine blood indexes. Therefore, the number of red blood cells, white blood cells, and blood platelets in the blood of postmenopausal rats and their morphological distribution can be detected through the blood analyzer to detect early signs of various clinical diseases (Lohsoonthorn, Dhanamun, & Williams, 2006). After the postmenopausal rats were given different doses of RS and fed with bean pulp, most of the indexes in the drug groups were not significantly different from those in the NC blank group, indicating that the rats would not have adverse reactions and effects due to the combined administration of RS and bean pulp (Table 3).

**TABLE 3:** Effect of RS‐mediated intestinal flora on the routine blood indexes of postmenopausal rats

indexes	NC	MC	RS	RSL	RSM	RSH	PC	FMT	Reference range
WBC (10^9^/L)	6.5 ± 1.2a	7.8 ± 1.4a	7.3 ± 1.2a	6.5 ± 1.4a	7.2 ± 1.4a	6.2 ± 2.1a	6.7 ± 1.1a	7.1 ± 1.2a	5.0–15.0
LYM (%)	22.5 ± 3.1d	24.5 ± 2.1cd	28.9 ± 3.1abc	27.6 ± 3.4bcd	33.8 ± 2.8a	31.2 ± 2.5ab	33.4 ± 3.4a	27.2 ± 2.6bcd	20.0–40.0
NEU (%)	52.4 ± 1.4d	51.4 ± 2.2d	60.5 ± 2.5abc	55.7 ± 3.2cd	62.8 ± 2.8ab	64.9 ± 1.9a	58.6 ± 4.3bc	52.3 ± 1.8d	50.0–70.0
MONO (%)	3.7 ± 0.6bc	4.2 ± 0.8abc	3.3 ± 0.5c	4.3 ± 0.2abc	4.6 ± 0.6ab	3.8 ± 0.5bc	5.1 ± 0.7a	3.7 ± 0.4bc	3.0–9.0
RBC (10^12^/L)	8.16 ± 1.1a	8.45 ± 1.4a	8.26 ± 0.9a	8.57 ± 1.5a	7.95 ± 0.8a	7.85 ± 0.6.a	8.34 ± 1.4a	7.49 ± 0.8a	7.2–9.6
HGB (g/L)	155.4 ± 11.7a	160.2 ± 15.6a	165.7 ± 21.4a	158.9 ± 16.8a	156.4 ± 18.7a	163.7 ± 22.5a	171.2 ± 17.3a	168.5 ± 20.4a	120–175
HCT (%)	0.44 ± 0.07a	0.37 ± 0.05a	0.42 ± 0.06a	0.45 ± 0.09a	0.38 ± 0.04a	0.41 ± 0.06a	0.35 ± 0.03a	0.43 ± 0.04a	0.35–0.49
MCV (FL)	59.4 ± 5.48ab	57.6 ± 6.12ab	56.8 ± 6.58ab	61.2 ± 4.76ab	53.6 ± 5.37b	62.4 ± 6.49ab	67.5 ± 5.34a	53.8 ± 6.42b	52.0–65.0
MCH (pg)	19.6 ± 1.2a	19.4 ± 1.4a	20.5 ± 2.1a	19.7 ± 1.6a	20.3 ± 1.4a	19.5 ± 1.5a	20.7 ± 1.3a	20.2 ± 2.2a	21.0–31.0
MCHC (g/L)	354.2 ± 33.2a	357.5 ± 38.5a	349.7 ± 34.6a	345.6 ± 42.7a	354.2 ± 45.6a	355.8 ± 39.4a	352.4 ± 38.5a	349.2 ± 43.7a	320–360
RDW‐*SD* (FL)	12.4 ± 1.1a	12.6 ± 1.5a	12.8 ± 1.4a	12.7 ± 1.5a	13.1 ± 1.7a	12.5 ± 0.9a	12.9 ± 1.6a	13.2 ± 1.5a	11.5–14.5
PDW (fL)	15.8 ± 2.2a	15.3 ± 2.6a	16.3 ± 1.7a	15.2 ± 2.5a	16.1 ± 2.3a	15.9 ± 1.7a	15.7 ± 2.1a	16.4 ± 2.5a	15.0–17.0
MPV (fL)	7.1 ± 0.5ab	7.2 ± 0.2ab	7.5 ± 0.3ab	7.9 ± 0.5a	6.9 ± 0.2b	7.6 ± 0.3ab	7.1 ± 0.5ab	7.3 ± 0.7ab	7.0–11.0
PCT (%)	0.15 ± 0.02ab	0.18 ± 0.05ab	0.16 ± 0.1ab	0.18 ± 0.08ab	0.21 ± 0.04ab	0.24 ± 0.05a	0.25 ± 0.03a	0.12 ± 0.06b	0.1–0.28

Note

Data are shown as mean ± *SD* (*n* = 7/9). Values with different letters in columns are significantly different (*p* < .05) from each other.

Lymphocytes (LYMs) originate from hematopoietic stem cells, circulate within the blood to the surrounding lymphoid organs, and then settle and proliferate within tissues. Under stimulation by certain antigens, lymphocytes can differentiate and multiply to produce effector cells, and perform immune functions (Aboulker, Autran, & Beldjord, 1992). Compared with the NC group, the LYMs of groups RSM, RSH, and PC were significantly different, which indicated that the specific immunity was stronger in the case of intragastric administration of the same amount of RS when administered in conjunction with bean pulp feed. MONO, the largest blood cell in the blood, is abundant in nonspecific lipase, which can resist and eliminate invading pathogenic microorganisms by means of phagocytosis and antibody production (Lin, Guo, & Yao, 2003). Compared with the NC group, the percentage of MONO in the PC group was slightly higher, indicating that the nonspecific immunity of the organism was stronger at this time. According to FMT, the immune performance of the body increased after transplanting the dominant bacteria producing equol, which was different from most of the indexes in the RS group, indicating that the immune activity of equol produced by RS‐mediated intestinal flora was higher than that of RS of equal dose.

### 3.4 Effect of RS on the intestinal flora structure of postmenopausal rats

**FIGURE 2:**
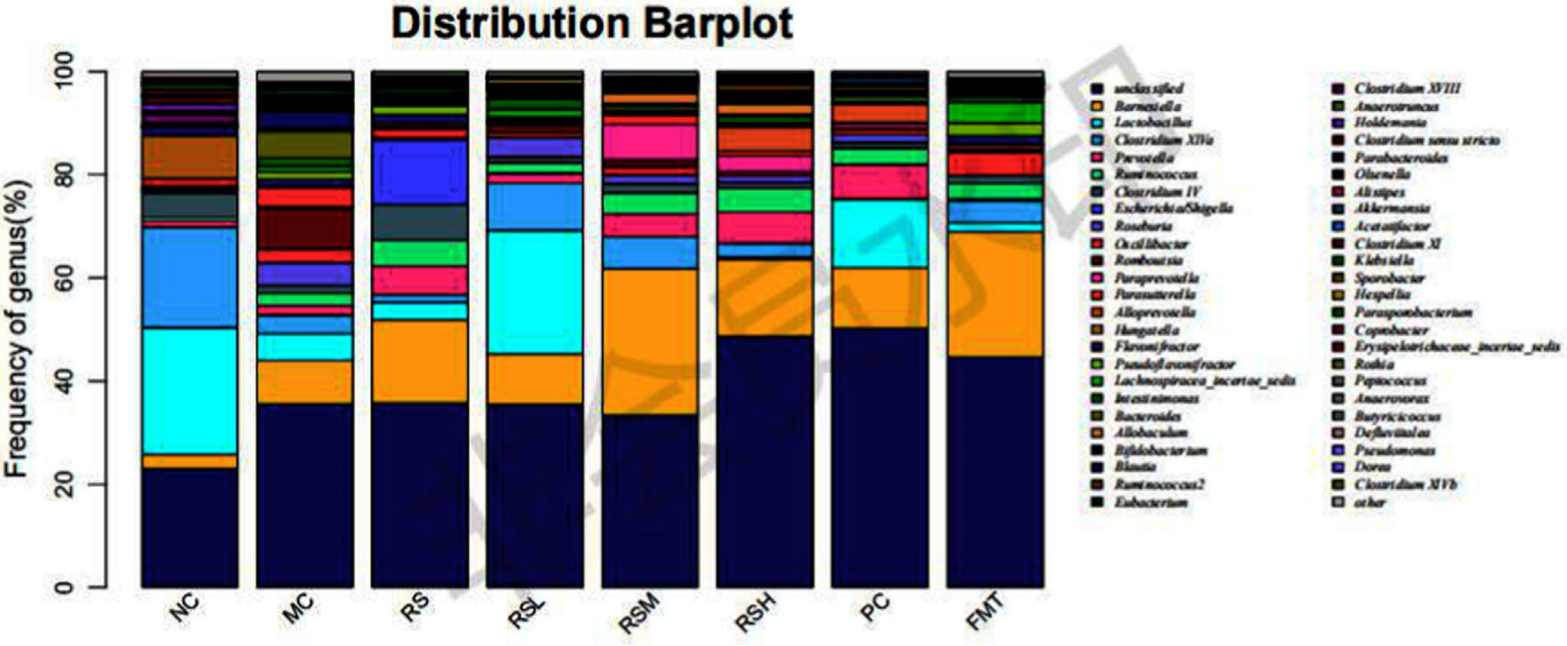
Effect of RS on the intestinal flora structure of postmenopausal rats. Note: 1 (uterus)A:NC B:MC C:RS D:RSH E:PC F:FMT； 1(C) 1(D) 1(E) 1(F) 2(A). Note: 2 (ovary)A:NC B:MC C:RS D:RSH E:PC F:FMT

The identification of the strains from the fecal samples of menopausal rats is shown in (Figure 2). The dominant bacteria in each experimental group were *Barnesiella*, *Lactobacillus*, *Clostridium* XIVa, *Prevotella*, *Ruminococcus*, and *Paraprevotella*. According to the 16S rDNA test results, compared with the NC group, *Romboutsia* genus in the MC group was more abundant and significantly different. Relevant studies have shown that this genus is a biomarker of early tumor formation (Mangifesta et al., 2018), indicating that postmenopausal rats see an increased risk of colon cancer (Li & He, 2013). After treatment, the drug group showed a significant decrease in the scope of this bacterial genus, indicating that both RS and equol indirectly produced by RS were associated with a reduced risk of colon and other cancers in postmenopausal rats; in the presence of soybean meal, the higher the concentration of resistant starch, the more significant the decline in this bacterial genus. *Lactobacillus* genus can synthesize and secrete lactic acid, which plays an important role in maintaining a healthy vaginal environment during childbearing years (Linhares, Summers, Larsen, Giraldo, & Witkin, 2011). The enrichment and growth of the *Lactobacillus* genus can form a community with social cohesion, increase the resistance against alien species, and constitute a biological barrier to maintain the health and microecological balance of the host reproductive tract (Huggins & Preti, 1976). After the Formestane‐based postmenopausal treatment, the richness of the *Lactobacillus* genus of the rats was significantly lower than that of the control group, indicating that the immunity of postmenopausal rats was reduced. The results of this study were consistent with the results of the routine blood test. After intragastric administration of RS and feeding of fodder containing bean pulp, the content of the *Lactobacillus* genus was increased, and the immune barrier of the rats was restored to a certain extent. The richness of the *Lactobacillus* genus in the intestinal flora of the rats after adding bean pulp was higher than that of the rats only receiving intragastric administration of RS, indicating that the effect of equol on enhancing the immunity of postmenopausal rats was stronger than that of RS. However, the FMT experimental group showed that the intestinal flora had the ability to convert daidzein from bean pulp into equol in the overall experimental process so as to reduce the risk of cancer and improve the recovery function in postmenopausal rats. Research has shown that *Lactobacillus* and *Clostridium* XIVa genera play an important role in the metabolism of daidzein in bean pulp (Decroos, Vanhemmens, & Cattoir, 2005; Schoefer, Mohan, Schwiertz, Braune, & Blaut, 2003). The richness of both genera in the RSL, RSM, and RSH experimental groups was higher than that in the RS group; in the RSL experimental group, the bacterial genera were richer than in the RSM and RSH groups. Therefore, this experiment further verified that *Lactobacillus* and *Clostridium* XIVa, as metabolic bacteria of daidzein, can convert daidzein into equol. That is, intestinal flora is the key factor in the regulation of the production of equol by RS.

### 3.5 Effect of RS‐mediated intestinal flora on the equol producing capability of postmenopausal rats

Changes in the contents of daidzein and equol in the urine of postmenopausal and experimental rats were detected by Elisa, and the logarithmic value of the ratio of equol and daidzein levels, namely log10 (equol/daidzein), was used to characterize the capability of individual rats to produce equol. After the successful establishment of the menopausal model at week zero, there was no significant difference in the capability to produce equol among the groups (Table 4). Since the rats were fed without bean pulp during the modeling period, there was no source of daidzein, or the content of equol was low; therefore, no significant difference between equol production among the groups was detected. After treatment of postmenopausal rats in each drug group, the log10 (equol/daidzein) values of all individuals were greater than −1.0, indicating highly efficient equol production. Therefore, it confirms that daidzein in bean pulp was the precursor of equol (Zheng, 2013). The FMT further showed that RS could transform daidzein into equol with higher biological function by regulating the composition of the intestinal flora and increasing the richness of lactobacillus and clostridium, playing an effective role as a phytoestrogen.

**TABLE 4:** Effect of RS on the equol producing capability of postmenopausal rats

week	NC	MC	RS	RSL	RSM	RSH	PC	FMT
0	0.42 ± 0.08a	0.50 ± 0.06a	0.38 ± 0.07a	0.45 ± 0.11a	0.42 ± 0.12a	0.46 ± 0.08a	0.47 ± 0.11a	0.41 ± 0.07a
1	1.48 ± 0.05a	1.55 ± 0.06a	0.41 ± 0.11b	1.48 ± 0.12ba	1.49 ± 0.08	1.43 ± 0.05a	1.55 ± 0.13a	1.39 ± 0.06a
2	1.39 ± 0.11bc	1.55 ± 0.09ab	0.41 ± 0.12d	1.29 ± 0.05c	1.69 ± 0.09a	1.55 ± 0.12ab	1.45 ± 0.07bc	1.48 ± 0.11b
3	1.39 ± 0.07b	1.64 ± 0.10a	0.32 ± 0.08c	1.52 ± 0.06ab	1.49 ± 0.11ab	1.65 ± 0.08a	1.51 ± 0.13ab	1.65 ± 0.05a
4	1.44 ± 0.11a	1.53 ± 0.08ab	0.34 ± 0.05c	1.62 ± 0.09a	1.55 ± 0.12ab	1.54 ± 0.09ab	1.53 ± 0.11ab	1.63 ± 0.07a
5	1.49 ± 0.12a	1.52 ± 0.08a	0.42 ± 0.05b	1.53 ± 0.13a	1.51 ± 0.11a	1.61 ± 0.09a	1.49 ± 0.07a	1.51 ± 0.05a
6	1.43 ± 0.08b	1.54 ± 0.12ab	0.34 ± 0.11c	1.54 ± 0.08ab	1.56 ± 0.06ab	1.65 ± 0.12a	1.44 ± 0.07b	1.58 ± 0.06ab

Note

Data are shown as mean ± *SD* (*n* = 7/9). Values with different letters in columns are significantly different (*p* < .05) from each other.

### 3.6 Effect of RS‐mediated intestinal flora on the morphology of the uterus and ovary of postmenopausal rats

**FIGURE 3:**
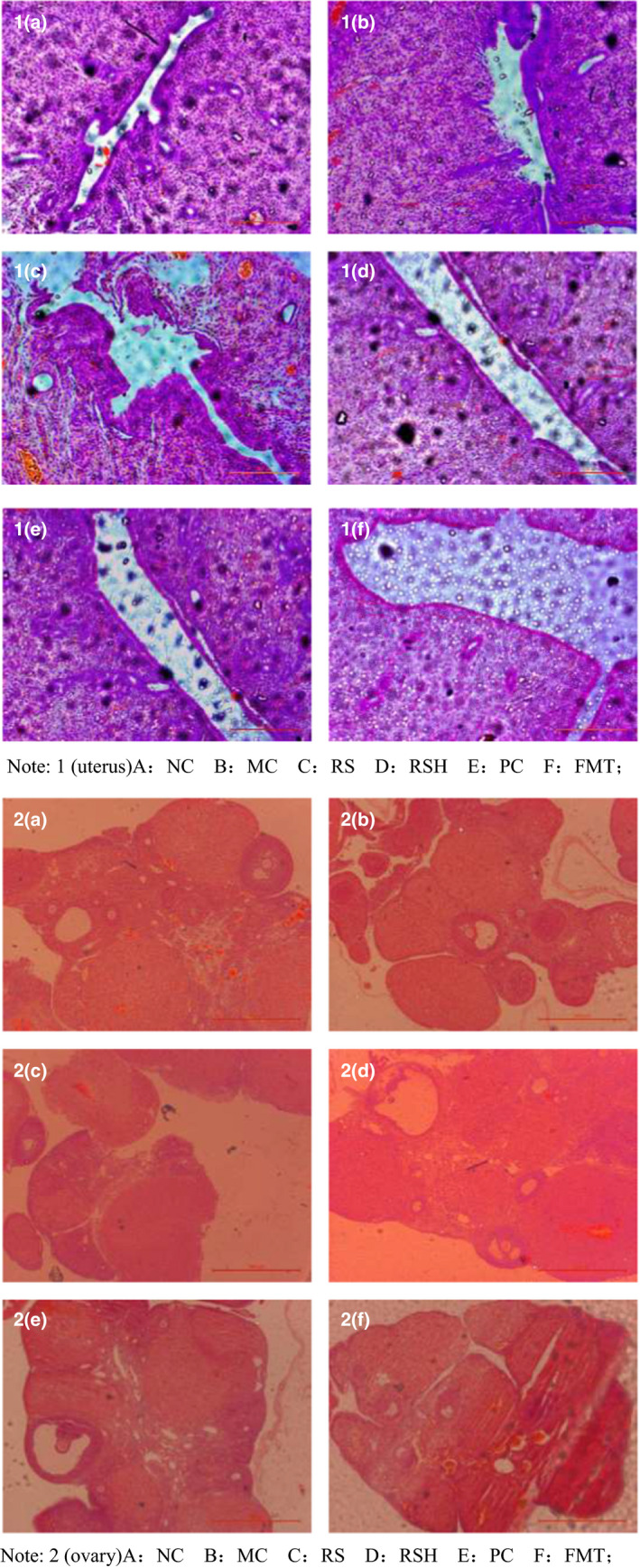
Effect of RS‐mediated intestinal flora on the morphology of the uterus and ovary of postmenopausal rats

The pathomorphological observation results of the uterus are shown in (Figure 3): group NC had thicker endometria, the glandular epithelium was of single layer and low columnar, the lamina propria was distributed with metrial glands and blood vessels, and the connective tissues were tight. Compared with the NC blank group, the MC group saw significantly reduced thickness of the uterine tube and the uterine mucosal epithelial cells. That is, the endometrium and muscle layer were significantly thinner, and the muscle fibers were small and loose. Through intragastric administration of RS in postmenopausal rats, endometrial atrophy was not changed in the RS group, and there were few intrinsic glands in the endometrium, indicating that simple intragastric administration of RS without bean pulp had no effect on the pathological phenomena of the uterus in postmenopausal rats. For the RSH group under the intervention of bean pulp, intragastric administration of RS could increase the thickness of the tube and the epithelium mucosae to a certain degree. The equol indirectly produced by RS can directly act on the uterus, promoting the growth of uterine epithelial cells, increasing the number of cells, blood vessels and glands, and relieving uterine contraction. No significant difference in improvement was detected compared to the PC group.

The morphological observation results of ovarian tissues in each group are shown in (Figure 3): the ovaries of the NC group or the young group were mature, with distinct layers of cortex and medulla, many growing follicles at different levels under light microscope including mature follicles, corpus luteum, and abundant follicular fluid (Huang, Zhang, & Zhang, 2002). In the MC group, the ovaries of postmenopausal rats were atrophied and poorly developed, with significantly reduced primordial follicles and growing follicles, irregular arrangement of granulosa cells, and apoptosis of corpus luteum cells. Through intragastric administration of RS, there was no significant difference in ovarian histological morphology between the RS group and the MC group, indicating that RS had no significant effect on ovarian atrophy. The experimental results of the RSH group showed that equol had a certain improvement effect on the ovaries of postmenopausal rats. Some tissues had mature follicles, and the total number of follicles was more than that in the MC group. Further, ovarian atrophy was greatly improved, and the improvement effect was not significantly different from that of the PC group. This indicated that with the intervention of bean pulp, RS indirectly converted daidzein into equol with higher estrogenic activities, thus relieving ovarian atrophy in postmenopausal rats. According to FMT, RS can improve the structure of intestinal flora and then convert daidzein from bean pulp into equol to mimic the role of estrogen.

## 4 CONCLUSIONS

In conclusion, this study elucidates the mechanism of RS‐mediated intestinal flora on the histological morphology of the uterus and ovaries of postmenopausal rats. This pertains to the observed phenomenon that organs, including the uterus, of postmenopausal women are characterized by functional decline. By comparing the atrophy incidence of reproductive target organs in rats, the correlation between RS and equol was established. Through the treatment of RS and feed of different compositions on rats, the improvement effect of equol on the reproductive organs of postmenopausal women was observed. Further, side effects of RS were verified from multiple perspectives such as the body weight of rats, efficiency of feed utilization, visceral indexes, routine blood indexes, and histological morphology of uterus and ovaries, and no detrimental effects were found. It is further proved that the simultaneous addition of resistant starch and soy products in the daily diet has a better repair effect on menopausal uterus and ovary than the consumption of resistant starch alone. Finally, the effects of RS on the intestinal flora of postmenopausal rats were determined through 16S rDNA, and this experiment further verified that *Lactobacillus* and *Clostridium* XIVa, as metabolic bacteria of daidzein, can convert daidzein into equol. That is, intestinal flora is the key factor in the regulation of the production of equol by RS.

## CONFLICT OF INTEREST

The authors declare that there are no conflicts of interest regarding the publication of this paper.
